# From Epistaxis to Embolization: A Case of Hereditary Hemorrhagic Telangiectasia Presenting With Spontaneous Hemothorax

**DOI:** 10.7759/cureus.88914

**Published:** 2025-07-28

**Authors:** VigneshKumar Kathiresan, Balamugesh Thangakunam

**Affiliations:** 1 Pulmonary Medicine, Christian Medical College Vellore, Ranipet Campus, Vellore, IND; 2 Pulmonary and Critical Care Medicine, Christian Medical College Vellore, Vellore, IND

**Keywords:** hematocrit, hemothorax, hht, pavm, pleural effusion

## Abstract

Hereditary hemorrhagic telangiectasia (HHT), or Rendu-Osler-Weber syndrome, is a rare autosomal dominant disorder characterized by mucocutaneous telangiectasias and visceral arteriovenous malformations (AVMs). We report a case of a 58-year-old female who presented with acute dyspnea and right-sided pleuritic chest pain. Her history included recurrent epistaxis since childhood and a positive family history of similar symptoms. Imaging revealed a right-sided hemorrhagic pleural effusion and pulmonary arteriovenous malformations (PAVMs), confirmed by CT pulmonary angiography and bubble contrast echocardiography. She underwent intercostal drainage followed by coil embolization of two large AVMs, with subsequent Amplatzer plug embolization on the contralateral side. Based on the Curacao criteria, a diagnosis of definite HHT was established. This case highlights hemothorax as a rare but potentially life-threatening initial manifestation of HHT. Early recognition, appropriate imaging, and timely embolization are essential to prevent recurrence and complications in patients with undiagnosed HHT presenting with pulmonary symptoms.

## Introduction

Hereditary hemorrhagic telangiectasia (HHT), also known as Rendu-Osler-Weber syndrome, is an autosomal dominant vascular disorder characterized by the presence of mucocutaneous telangiectasias and arteriovenous malformations (AVM) in various organs, including the lungs, liver, brain, and gastrointestinal tract [1]. It is attributed to mutations in genes associated with the transforming growth factor-beta (TGF-β) signaling pathway, notably ENG and ACVRL1. Although recurrent epistaxis is the most frequently observed symptom, visceral AVMs, particularly pulmonary arteriovenous malformations (PAVMs), can precipitate severe complications such as hypoxemia, stroke due to paradoxical embolism, and hemorrhage [2]. Hemothorax resulting from the rupture of a PAVM is an infrequent initial presentation [3]. In this report we detail a case of an elderly female patient with HHT who developed hemothorax due to a ruptured PAVM. This case is noteworthy for highlighting the diagnostic challenge in recognizing hemothorax when conventional criteria are not met and emphasises the role of embolization in effective management.

## Case presentation

A female patient in her late 50s presented to our emergency department with dyspnea persisting for one week, accompanied by right-sided pleuritic chest pain that commenced 10 days prior to her presentation. She had previously sought medical attention at a local hospital, where laboratory investigations revealed a hemoglobin level of 3 mg/dL. Consequently, she received a transfusion of three units of packed red blood cells before presenting to our facility. Her past medical history disclosed recurrent spontaneous episodes of epistaxis since childhood, which typically resolved upon applying nasal pressure for 10-15 minutes. Her family history was notable for similar episodes of epistaxis affecting her father, two siblings, and daughter.

**Figure 1:**
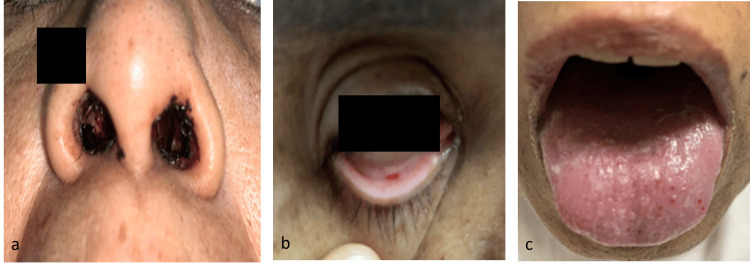
Showing clotted blood in the anterior nares suggestive of recent epistaxis (a), telangiectasia in the palpebral conjunctiva (b) and tongue (c) Written informed consent for publication of identifiable images was obtained from the patient and submitted to the journal.

Upon presentation to the emergency department, the patient was tachypneic with a respiratory rate of 36 breaths per minute, a pulse rate of 99 beats per minute, blood pressure measuring 140/60 mmHg, and an oxygen saturation (SpO2) of 89% on room air, which improved to 92% with the administration of 2 L/min of oxygen. The patient appeared pale, with clotted blood staining of the anterior nares and telangiectasia on the tongue and palpebral conjunctiva (Figure 1).

Chest auscultation revealed absent breath sounds in the right mid and lower hemithorax. Examination of the abdomen, the cardiovascular and central nervous systems, was unremarkable.

**Figure 2:**
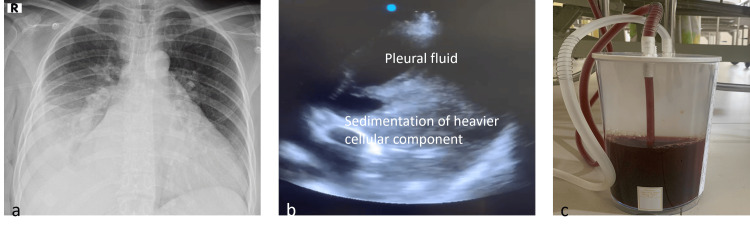
Chest radiography suggestive of right moderate pleural effusion (a), Ultrasound of the chest demonstrating the hematocrit sign, seen as fluid layering caused by sedimentation of red blood cells, suggestive of hemothorax. (b), Spictra showing haemorrhagic drain from the intercostal drainage tube (c).

Blood tests indicated a hemoglobin level of 10.4 mg/dL (after three transfusions received before arriving at our facility). Arterial blood gas analysis revealed respiratory alkalosis with an alveolar-arterial (A-a) difference of 50 mmHg. The patient's coagulation profile was within normal limits. A chest radiograph suggested a moderate right pleural effusion (Figure 2A). Ultrasound examination of the chest identified a moderate pleural effusion with two distinct echogenicities, indicative of the hematocrit sign (Figure 2B). Diagnostic pleural aspiration revealed a hemorrhagic effusion with a red blood cell count of 7.0x105 cells/mm3, predominantly neutrophilic (81%), and exudative in nature.

**Figure 3:**
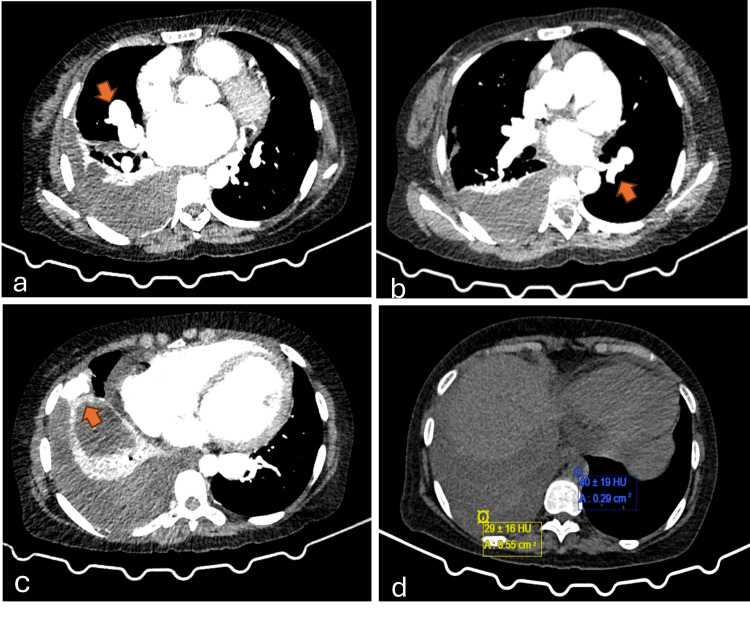
CTPA showing dilated and tortuous pulmonary vessel (arrow) (a), anomalous communication between left pulmonary vein and branch of left pulmonary artery (b), dilated subpleurally located dilated pulmonary vessel (arrow) (c), Non-contrast CT of thorax- Ratio of attenuation values in pleural fluid and aortic blood of 0.72 (threshold: ≥0.56). CTPA: computed tomography pulmonary angiography, HU: Hounsfield units.

The pleural fluid to blood hematocrit ratio was 21.2. Computed tomography pulmonary angiography (CTPA) demonstrated a moderate hyperdense pleural effusion on the right side, a grossly dilated and tortuous pulmonary vein on the right side subpleurally, and an anomalous communication between the segmental branch of the left pulmonary artery and the left inferior pulmonary vein (Figure 3A-3C). On non-contrast computed tomography (NCCT) of the chest, the pleural fluid exhibited an attenuation of 29 Hounsfield units (HU) (threshold of ≥15.6 HU). Additionally, the pleural fluid-to-aortic blood attenuation ratio (PF/AB) was 0.72, surpassing the cutoff of ≥0.56, supporting the diagnosis of hemothorax despite a pleural fluid hematocrit below 50% (Figure 3D).

Bubble contrast echocardiography showed dense left heart opacification via the pulmonary veins, occurring four cycles after complete right heart opacification, suggestive of a significant extracardiac shunt. The patient's clinical presentation met Curacao's criteria for HHT, including recurrent epistaxis, visceral AVM, multiple telangiectasias, and a family history indicative of HHT in first-degree relatives.

**Figure 4:**
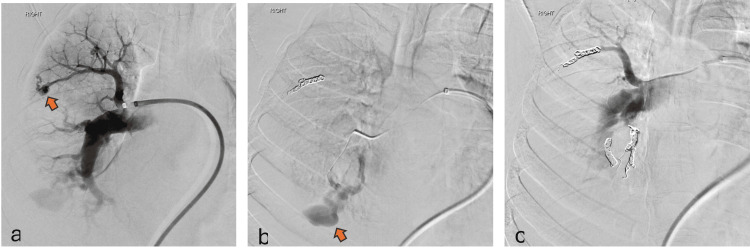
Pulmonary angiography showing two large aneurysms pre-embolization(arrows) (a and b) and successful occlusion post-coiling (c), the gold-standard treatment.

She underwent intercostal drainage (ICD) tube insertion for the right hemothorax. Subsequently, pulmonary angiography was performed, which showed multiple arteriovenous fistulae (AVF) followed by endovascular coil embolization of the two large AVF (Figure 4).

Once the ICD drain was reduced, the tube was removed, and there was no further reaccumulation. One year later, she underwent endovascular embolization of the AVF on the left side using two 8 mm Amplatzer vascular plugs. Brain magnetic resonance venography (MRV) was performed as part of the HHT diagnostic evaluation, which revealed no AVM. Gastroscopy was normal and colonoscopy was recommended; however, the patient declined to undergo this procedure. The patient was initiated on thalidomide 50 mg once daily for the management of recurrent epistaxis and advised to take tranexamic acid 500 mg as needed during episodes of epistaxis.

## Discussion

HHT is a disorder characterized by abnormal blood vessel formation, following an autosomal dominant inheritance pattern with variable penetrance [2]. The initial description of the disease manifestations was provided by Benjamin Guy Babington in 1865 and John Wickham Legg in 1876 [2]. Subsequently, in 1886, Henri Rendu identified an association between epistaxis, cutaneous telangiectasia, and familial occurrence. This condition was further delineated through individual contributions by Sir William Osler in 1901 and F. P. Weber in 1907. These foundational contributions led to the eponymous recognition of the disease as Rendu-Osler-Weber syndrome [4]. It is among the most prevalent monogenic disorders, with a global incidence ranging from one in 5,000 to one in 10,000. There are significant geographic variations in its prevalence, with notably high rates observed in the French Department of Ain, Vermont, and the Netherlands Antilles [2].

The etiology of HHT is attributed to mutations in genes associated with the TGF-β signaling pathway, leading to the development of abnormal vascular structures such as telangiectasias and visceral AVMs [4]. The primary genes implicated in HHT include endoglin (ENG) located on chromosome 9q, accounting for 39-59% of cases, and the activin receptor-like kinase 1 (ACVRL1, ALK-1) gene on chromosome 12q, responsible for 25-57% of cases. Both genes encode proteins essential for the TGF-β signaling pathway. Less frequently, mutations occur in SMAD4 on chromosome 18q (1-2%), GDF2 on chromosome 10q (<1%), as well as in HHT3 and HHT4 [5].

The clinical manifestations of the condition become more pronounced with advancing age due to late-onset penetrance [2]. Although symptoms frequently emerge before the age of 20, the complete diagnostic criteria are typically satisfied after the age of 40 years [6]. The diagnosis of HHT was primarily determined using the Curacao criteria, which were developed by the Scientific Advisory Board of the HHT Foundation International, Inc. [7]. These criteria include spontaneous and recurrent epistaxis; multiple telangiectasia located at characteristic sites such as the lips, oral cavity, fingers, and nose; visceral AVMs involving the lungs, gastrointestinal tract (with or without bleeding), liver, brain, or spine; and a first-degree relative meeting the criteria for HHT. The presence of at least three of the four criteria confirms the diagnosis of definite HHT. Conversely, the presence of fewer than two criteria renders the diagnosis of HHT unlikely. Nevertheless, children of affected individuals remain at risk due to the late-onset penetrance of this disorder [7]. Our patient met all four criteria, confirming a diagnosis of definite HHT.

The prevalence of PAVM in HHT is estimated to be approximately 15-35% [8]. Notably, 70-80% of PAVMs occur in association with HHT. In cases where multiple PAVMs are present, there is a 90% likelihood that the patient will have HHT. PAVMs have the potential to rupture into the bronchial tree or the pleura. When a PAVM is located peripherally near the visceral pleura, hemothorax is more prevalent, whereas proximity to the bronchial tree increases the likelihood of hemoptysis [9]. In our patient, the PAVM situated closer to the visceral pleura was identified as the cause of the hemothorax. In a case series of 143 patients with HHT by Ference et al., 8% experienced severe hemorrhagic manifestations, with nine out of 11 cases being the inaugural presentation and hemothorax was reported in 3% of patients with PAVM [10]. In a multicenter retrospective study by Cottin et al. also reported hemothorax in four cases (3%) in their series of 126 patients. The traditional risk factors for the rupture include pregnancy, especially in the second and third trimesters, pulmonary arterial hypertension, advanced age and use of anticoagulation [5,8,11]. Our patient did not have any of the risk factors at the time of developing hemothorax.

While hemothorax is conventionally diagnosed when the pleural fluid hematocrit exceeds 50% of the blood hematocrit, our patient demonstrated a pleural fluid to serum hematocrit ratio of 21.2, which does not fulfill the criteria for hemothorax. This reduced hematocrit ratio may result from secondary dilution and pleural fluid layering, as suggested by the hematocrit sign on chest ultrasound [12,13]. According to Liu et al., a threshold of ≥15.6 HU demonstrated a sensitivity of 86.8% and a specificity of 97.4% in distinguishing pleural effusion from hemothorax. Additionally, an attenuation ratio of PF/AB of ≥0.56 exhibited a sensitivity of 76.3% and a specificity of 90.4% [14]. In our case, the pleural fluid exhibited an attenuation of 29 HU and a PF/AB ratio of 0.72, indicative of hemothorax (Figure 3D). Furthermore, the patient presented to our facility 10 days post-symptom onset and had received three units of blood transfusions prior to arrival.

CTPA remains the definitive method for visualizing the anatomy of PAVM, effectively illustrating the feeding vessels, draining veins, and the aneurysm responsible for hemorrhagic complications. The presence of an "anomalous bulge sign" on CTPA and a "double shadow sign" on digital subtraction angiography is indicative of a PAVM rupture [15]. The management of PAVM in HHT aims to prevent further growth or hemorrhagic complications resulting from rupture. The primary treatment modalities include transcatheter embolization and surgical intervention.

Transcatheter embolization with coils has emerged as the primary treatment modality for PAVM, supplanting the need for surgical intervention in most instances with a success rate of 85-98% [2,9]. Amplatzer vascular plugs are increasingly favored over coils due to their capacity to occlude large feeding vessels with a single plug, thereby reducing both procedural duration and radiation exposure [1]. Embolization is indicated even in asymptomatic patients with feeding vessels exceeding 3 mm in diameter to prevent complications such as paradoxical embolism and hemorrhage [11]. In our patient, following the insertion of an ICD, transcatheter embolization with coiling was performed, resulting in the resolution of hemothorax and no further recurrence. The PAVM on the contralateral side was occluded with Amplatzer vascular plugs one year after the initial procedure. Surgical resection of the PAVM, involving lobectomy or wedge resection, is typically reserved for emergencies, embolization failure, or cases deemed unsuitable for embolization [2,3]. Lung transplantation is considered in rare cases where the PAVMs are diffuse and severe [2].

## Conclusions

This case underscores hemothorax as an uncommon (3-4%) but potentially life-threatening manifestation of HHT. Although pleural fluid hematocrit was below the conventional threshold, clinical context and imaging findings were essential in recognizing hemothorax, highlighting that traditional criteria may not always be fulfilled, especially in late presentations like this case. This emphasizes the importance of maintaining heightened suspicion in patients with epistaxis and hypoxemia. Timely embolization despite staged intervention - initial coil embolization followed by Amplatzer plug placement a year later - prevented recurrence, reinforcing its role as first-line therapy. While genetic testing could not be performed in this case, adherence to the Curacao criteria enabled a confident diagnosis of HHT in this patient. 
